# Nephropexy

**Published:** 1915-10

**Authors:** 


**Nephropexy.** Marion, Jour. d'Urologie advocates a revival of this operation. He reports 53 operations without deaths. 26 cases were followed. Only one case was made worse. Pain was relieved. Digestive disturbances were cured in 6 to 16 patients. 4 nervous conditions were relieved out of 8. (Note: We confess to a prejudice against this operation. E.g. woman, 3d degree right kidney, operated on contrary to advice. Kidney firm as a rock, 10 years after operation, in a peculiar slanting abnormal position. Almost constant pain. Similar case, kidney fairly firm but urine has shown albumin and casts after operation, normal previously. Case of unexplained nephralgia. Kidneys not movable but anchored under belief that they were. Pain not relieved. Case referred back on the ground that kidneys had worked loose again. They were not loose and never had been. On the other hand, there is no question but that certain cases are permanently relieved by operation. Still, a bandage holds the kidneys in fairly good position and relieves the pain and nervous reflexes. Slight movability may be permanently cured by deposit of fat due to better nutrition. Third and usually second degree cases cannot be cured by medical treatment but, after wearing the bandage for a few months, permanent subjective relief may usually be expected and, at the worst, the bandage has to be resumed occasionally.

